# Activation of Human Salivary Aldehyde Dehydrogenase by Sulforaphane: Mechanism and Significance

**DOI:** 10.1371/journal.pone.0168463

**Published:** 2016-12-20

**Authors:** Md. Fazle Alam, Amaj Ahmed Laskar, Lubna Maryam, Hina Younus

**Affiliations:** Enzymology Laboratory, Interdisciplinary Biotechnology Unit, Aligarh Muslim University, Aligarh, India; Islamic Azad University Mashhad Branch, ISLAMIC REPUBLIC OF IRAN

## Abstract

Cruciferous vegetables contain the bio-active compound sulforaphane (SF) which has been reported to protect individuals against various diseases by a number of mechanisms, including activation of the phase II detoxification enzymes. In this study, we show that the extracts of five cruciferous vegetables that we commonly consume and SF activate human salivary aldehyde dehydrogenase (hsALDH), which is a very important detoxifying enzyme in the mouth. Maximum activation was observed at 1 μg/ml of cabbage extract with 2.6 fold increase in the activity. There was a ~1.9 fold increase in the activity of hsALDH at SF concentration of ≥ 100 nM. The concentration of SF at half the maximum response (EC_50_ value) was determined to be 52 ± 2 nM. There was an increase in the *V*_*max*_ and a decrease in the *K*_*m*_ of the enzyme in the presence of SF. Hence, SF interacts with the enzyme and increases its affinity for the substrate. UV absorbance, fluorescence and CD studies revealed that SF binds to hsALDH and does not disrupt its native structure. SF binds with the enzyme with a binding constant of 1.23 x 10^7^ M^-1^. There is one binding site on hsALDH for SF, and the thermodynamic parameters indicate the formation of a spontaneous strong complex between the two. Molecular docking analysis depicted that SF fits into the active site of ALDH3A1, and facilitates the catalytic mechanism of the enzyme. SF being an antioxidant, is very likely to protect the catalytic Cys 243 residue from oxidation, which leads to the increase in the catalytic efficiency and hence the activation of the enzyme. Further, hsALDH which is virtually inactive towards acetaldehyde exhibited significant activity towards it in the presence of SF. It is therefore very likely that consumption of large quantities of cruciferous vegetables or SF supplements, through their activating effect on hsALDH can protect individuals who are alcohol intolerant against acetaldehyde toxicity and also lower the risk of oral cancer development.

## Introduction

The aldehyde dehydogenase (ALDH) superfamily consists of 19 isozymes, which catalyze the oxidation of endogenous and exogenous toxic aldehydes (short, long aliphatic and aromatic) into non-toxic corresponding acids [1,2]. Modest consumption of ethanol is believed to be beneficial for health, whereas large consumption can cause several complications [3,4]. Ethanol is first oxidized by alcohol dehydrogenase into the primary metabolite acetaldehyde, which is mutagenic and placed in group 1 carcinogens [5]. ALDH than detoxifies acetaldehyde by oxidizing it to acetic acid. More than 40% East Asian population (~ 560 million or ~ 8% of the world population) cannot oxidize the toxic acetaldehyde into the non-toxic acid because of having a point mutation in the ALDH2 gene which results in almost no enzyme activity, leading to the accumulation of the toxic acetaldehyde [6–8]. ALDH2 deficient people are very sensitive to alcohol, a small dose of it can cause severe consequences like nausea, facial flushing, tachycardia and long-lasting headache [9]. There are many reports indicating the chances of occurrence of malignancy and other serious health problems due to ALDH polymorphism [10]. Particularly, the occurrence of squamous cell carcinoma in the upper aerodigestive track (UADT) among the middle aged East Asian populations is associated with the ALDH2 polymorphism [6,11]. There is more than 80 fold increased risk for squamous cell carcinomas in the UADT of heavy drinker heterozygotic individual (ALDH*1/*2) compared with only about 4 fold increase in the wild-type (ALDH*1/*1) heavy drinkers [6,12–14].

Human salivary aldehyde dehydrogenase (hsALDH) found in the saliva is basically ALDH3 isoform of the ALDH enzyme. ALDH3 is highly expressed in the epithelial lining of the UADT, kidney, mammary gland, liver and stomach [15,16]. HsALDH catalyzes the oxidation of the aromatic aldehydes having bulky side chains, medium/long chain aliphatic aldehydes and α,β-hydroxyalkenal aldehydes, but not acetaldehyde under basal condition [17]. Moderate consumption of ethanol by ALDH2*1/*2 heterozygote individuals leads to a significant increase in the acetaldehyde concentration in the saliva which forms an adduct interacting with DNA, resulting in severe health issues [18]. There are reports on strategies to increase the rate of acetaldehyde elimination especially in ALDH2*1/*2 heterozygotes through the modulation of the activity of ALDH2 by chemical chaperones, and by inducing and recruiting other ALDH isoforms [4,19,20].

The cruciferous vegetables which include broccoli, cauliflower, radish, kale, cabbage and brussels sprouts are a rich source of health beneficial secondary metabolites [21]. Much of the health benefits have been attributed to the physiological effects of the isothiocyanates, especially sulforaphane (SF) which has been shown to protect against various types of cancers, diabetes, atherosclerosis, respiratory diseases, cardiovascular diseases, neurodegenerative disorders and ocular disorders [22,23]. A number of mechanisms by which SF protects cells have been reported which includes induction of apoptosis, cell cycle arrest, anti-inflammatory effects, inhibition of phase 1 (cytochrome P450) enzymes and induction of phase 2 detoxification enzymes [23–25]. Therefore, modulation of important enzymes like quinone reductase, glutathione *S*-transferase, ALDHs and ribonucleoside diphosphate reductase by natural bioactive compounds like SF or synthetic chemical compounds may be a significant component of their anticarcinogenic action [26,27]. Recently it has been shown that SF accelerates acetaldehyde metabolism by inducing ALDHs and hence may protect individuals who are alcohol intolerant against acetaldehyde toxicity [20].

The present study was undertaken after getting very encouraging results from one of our previous studies where a chemical activator or chaperone (Alda 1) was designed which activated ALDH2 and restored near wild type activity of ALDH2*2 [4]. In this study, we aimed to investigate the effect of different types of cruciferous vegetables extracts and their bio-active compound SF on the activity of hsALDH. Since they activated this very useful detoxifying enzyme, we than evaluated the mechanism of activation of the enzyme by SF through kinetic measurements and different biophysical techniques. Molecular docking analysis was done to determine the binding site and the amino acid residues of the enzyme involved in the interaction with SF.

## Materials and Methods

### Materials

SF, 6-methoxy-2-naphthaldehyde, NAD^+^, NADH, DTT and acetaldehyde were purchased from Sigma. Acetone, ethanol, acetonitrile, n-hexane and chloroform were obtained from SRL, India. Fresh cruciferous vegetables like cabbage, cauliflower, radish, turnip and broccoli were purchased from the local market of Medical Road, Aligarh, India in the month of November, 2015. These vegetables are grown in the agricultural field of the locality and made available to us through local vendors. EDTA was the product of Himedia chemicals, India. All other chemicals and reagents used were of analytical grade.

### Preparation of crude extract of cruciferous vegetables

Fresh broccoli, cabbage, cauliflower, turnip and radish were obtained. The edible parts were collected in each case, washed and air dried in portions. The dried portions were combined and homogenized in 50 ml methylene chloride for 5 min. The crushed homogenate was left to autolyze at room temperature for 5 min. After autolysis, the homogenate was filtered and again extracted with 50 ml methylene chloride. The extracted fractions were combined and salted with anhydrous 5 mM sodium sulphate. The methylene chloride fractions were dried at 30°C under vacuum on a rotatory evaporator. The residue was dissolved in acetonitrile and then filtered through a 0.22 μm filter and stored for further use.

### HsALDH activity measurements

Preparation of crude saliva and purification of hsALDH by DEAE-Cellulose column was carried out according to our published procedure [28]. The collection of human saliva samples was approved by the institutional ethical committee of Interdisciplinary Biotechnology Unit (IBU), Aligarh Muslim University, Aligarh, India. The participants were the research students of IBU (MFA, AAL, LM) who gave a verbal consent to their research supervisor (HY) and the members of the ethical committee, which was approved. The activity assay of crude and pure hsALDH (ALDH3A1) was done using fluorogenic naphthaldehyde substrate. In particular, 6-methoxy-2-naphthaldehyde is used to measure selectively the activity of the ALDH3A1 isoform [29]. All the activity assays were performed in 50 mM sodium phosphate buffer, pH 7.5 at 25°C, in the presence of 0.5 mM DTT and 0.5 mM EDTA. Substrate, 6-methoxy-2-naphthaldehyde (5 μM) and coenzyme NAD^+^ (100 μM) were used to measure the activity of hsALDH [30,31]. The reaction was started by the addition of the enzyme at 25°C and monitoring continuously for 5 min. Fluorescence background noise, if any, was measured prior to enzyme addition and subtracted from the final slope. Fluorimetric assays were run on a Shimadzu RF-5301PC instrument with excitation and emission wavelengths as 315 and 360 nm, respectively. The activity of hsALDH was expressed in terms of nanomolar (nM) concentration of flourogenic product or (NADH) formation per min per μg of the enzyme under the standard assay conditions using a standard plot of the respective product.

### Effect of SF on the activity of hsALDH

The effect of the different vegetable extracts and SF on the activity of purified hsALDH was studied by incubating the enzyme for 1 min in presence of different concentration of the vegetable extracts (0–2.5 μg/ml) or SF (0–500 nM). Activity was then determined under standard assay condition as described above. Data were fitted in the case of SF into non-linear regression analysis curve of log (SF) vs activity to determine the EC_50_ value under “Dose response Stimulation” using “Graphpad Prism 6.0”.

### Substrate dependent activity assay in absence/presence of SF

The activity of hsALDH was determined by varying the concentration of the substrate (0–20 μM) in absence and presence of 50 nM SF at standard reaction conditions. All the apparent enzyme kinetic parameters (*V*_*max*_ and *K*_*m*_) were determined by fitting the data in non-linear regression analysis curve of Michaelis-Menten plot and Line-weaver Burk plot under ‘Enzyme kinetic’ function in “Graphpad Prism 6.0”.

### UV absorption analysis

Absorption measurements were performed using a Perkin-Elmer (Lambda 25) double beam UV-VIS Spectrophotometer. Fixed concentration of hsALDH (0.2 mg/ml) and varying concentration of SF (0–300 nM) were taken and spectra were recorded from 250–350 nm at 25°C.

### Fluorescence quenching measurements

Fluorescence quenching measurements were done on a Shimadzu 5301 PC fluorescence spectrophotometer equipped with a constant temperature holder and the temperatures were maintained by a constant temperature water circulator (Julabo Eyela). The excitation and emission slit widths were set at 3 nm. Titration of SF (0–60 nM) with hsALDH solutions (0.2 mg/ml) was carried out in a dual-path length fluorescence cuvette (10 x 3.5 mm). Intrinsic fluorescence was measured by exciting the sample at 280 nm. The emission spectra were recorded in the range of 300–450 nm and the data were plotted by taking the emission intensity at 339 nm. The decrease in fluorescence intensity at 339 nm was analyzed using the following Stern-Volmer Eq (1):

$${\text{F}}_{\text{o}}/\text{F}={\text{K}}_{\text{sv}}[\text{Q}]+1=\text{Kq}\tau_{\text{o}}[\text{Q}]+1$$ (1)

where, F_0_ and F are fluorescence intensities in absence and presence of SF, K_sv_ is the Stern-Volmer quenching constant, K_q_ is the bimolecular rate constant of the quenching reaction and τ_0_ is the average integral fluorescence life time of tryptophan, which is ~10^−9^ s. For the correction of inner filter effect of protein and ligand we used following Eq (2) [32,33]:

$${\text{F}}_{\text{cor}}={\text{F}}_{\text{obs}}10^{({\text{A}}_{\text{ex}}+{\text{A}}_{\text{em}})/2}$$ (2)

Where F_cor_ and F_obs_ are corrected and observed fluorescence intensity, Aex and Aem are the absorption of the system at excitation (280 nm) and emission (339 nm) wavelength, respectively. Binding constant and number of binding sites were obtained from the following modified Stern-Volmer Eq (3):

$$\text{log}({\text{F}}_{\text{o}}/\text{F}-1)=\text{log }{\text{K}}_{\text{b}}+\text{n log}[\text{Q}]$$ (3)

Where, K_b_ is binding constant and n is number of binding sites.

### Far UV-Circular Dichroism (CD) measurements

The CD studies of hsALDH in the absence and presence of SF were carried out with a JASCO-J815 Spectropolarimeter equipped with a Peltier-type temperature controller. The instrument was calibrated with D-10-camphorsulfonic acid. All the CD measurements were performed at 25°C. Spectra were collected with a 50 nm min^-1^ scan speed, 0.1 nm data pitch and a response time of 2 s. Each spectrum was the average of 2 scans. The far UV-CD spectra were recorded in the wavelength range of 190–250 nm. All the spectra were smoothed by the Savitzky-Golay method with 25 convolution width. 0.2 mg/ml hsALDH concentration was used for the experiment.

### Effect of SF on the activity of hsALDH towards acetaldehyde

The activity of hsALDH towards acetaldehyde in the absence and presence of SF was determined using the above standard activity assay method using acetaldehyde (50 μM) as the substrate. NADH fluorescence was measured to determine the activity in terms of NADH formation. The instrumental settings for NADH were: Excitation at 340 nm and emission at 460 nm, with spectral bandwidths of 10 nm for both excitation and emission beams. The amount of NADH formed was determined from the standard curve of NADH at 460 nm.

### Molecular docking studies

To determine the binding site and the amino acid residues of ALDH3A1 interacting with SF, *in silico* docking studies were performed by AutoDock Vina (http://vina.scripps.edu/) and further confirmed by commercially available docking software GOLD. The crystal structure of apoform of ALDH3A1 was obtained from Protein Data Bank (PDB ID: 3SZA), and sdf file of SF (CID: 5350) was obtained from PubChem database. Docking analysis was carried out with the grid size set as 60, 60 and 60 along the X, Y and Z axes with 0.375 Angstrom grid spacing. The 10 best solutions based on docking score were retained for further analysis. Discovery studio 3.5 was used for visualization and for the identification of residues involved in binding.

## Results and Discussion

SF derived from its glucosinolate precursor contained in cruciferous vegetables has been reported to increase the phase II antioxidant enzymes in the human upper airway and has recently been shown to induce ALDHs [20,34]. The level of hsALDH was found to be high in the saliva of subjects who continuously ingest large quantities of broccoli [16]. Therefore, in the present study, the direct effect of different cruciferous vegetable extracts and SF on the activity of pure hsALDH has been determined.

### Effect of different cruciferous vegetable extracts on the activity of hsALDH

**Fig 1:**
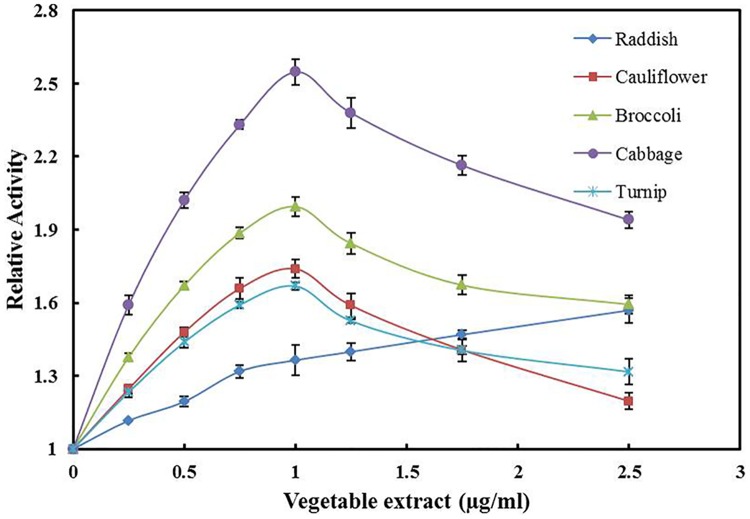
Effect of cruciferous vegetable extracts on the activity of hsALDH. The amount of hsALDH used in the reaction was 20 μg, concentration of the substrate (6-methoxy-2-naphthaldehyde) was 5 μM, concentration of NAD was 100 μM and the reaction time was 5 min. Each point represents the mean of two experiments carried out in triplicates.

The effect of five common cruciferous vegetables on the activity of hsALDH has been studied. The relative activity of the enzyme in the presence of varying concentration of each vegetable extract is shown in Fig 1. Each of the vegetable extract in the concentration used (till 2.5 μg/ml) activated the enzyme. Maximum activation was observed at 1 μg/ml of cabbage, broccoli, cauliflower and turnip extract with 2.6, 2.0, 1.7 and 1.6 fold increases in activity, respectively. For these vegetable extracts, when the concentration of the extract was increased above 1 μg/ml, the activating effect decreased gradually. However, the enzyme activity still remained higher than that in the absence of the vegetable extract even at 2.5 μg/ml of the extract. For the radish extract in the concentration range used, the relative activity kept increasing with increasing concentration of the extract. There was 1.3 fold increase in the activity at 1 μg/ml of the extract. Therefore, it is evident from the data that out of all the five cruciferous vegetables used in this study, cabbage exhibited the maximum activating effect on the activity of hsALDH. The level of glucoraphanin from which SF is derived is highly variable in different cruciferous vegetables and broccoli is a rich source of glucoraphanin [35]. However, still higher amounts are present in some cultivars of cabbage [36]. Therefore, the higher level of SF in cabbage extract may be the reason why this extract exhibited the maximum activating effect on hsALDH activity.

### Effect of SF on the activity of hsALDH

**Fig 2:**
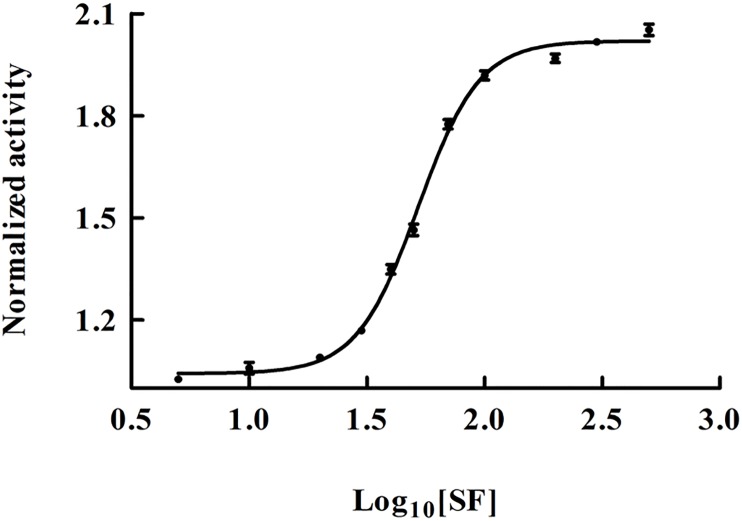
Dose response curve of SF. HsALDH activity was determined in presence of 0–500 nM concentration of SF using 15 μg of the enzyme in 1 ml of total reaction mixture. Each point represents the mean of two experiments carried out in triplicates.

The effect of SF on the activity of hsALDH was investigated. It was found that the activity of hsALDH remained the same till 20 nM concentration of SF (Fig 2). However, with further increase in the concentration of SF, the activity of the enzyme increased gradually upto 100 nM concentration of SF, after which the activity remained constant till 500 nM concentration of SF (Fig 2). With further increase in the concentration of SF (above 500 nM), there was a slight decrease in the enzyme activity which may be due to molecular crowding or non-specific binding of SF to the enzyme (data not shown). However, even at these high concentrations of SF examined, the activity was still higher than that in the absence of SF. Therefore, SF exerted an overall activating effect on the enzyme. The activity of hsALDH was increased by about 1.9 fold in presence of 100 nM SF. The concentration of SF at half maximum response (EC_50_ value) was determined to be 52 ± 2 nM. Therefore, SF increases the activity of hsALDH to a good extent. However, crude cruciferous vegetables exhibited a greater stimulating effect on the activity of the enzyme than SF alone. This might be because of the presence of other beneficial compounds other than SF that shows cumulative effect to activate the enzyme. Ushida and Talalay (2013) similarly observed that out of the 20 compounds they examined, 15 of them including isothiocyanates such as SF, flavonoids, terpenoids, etc. increased the total ALDH specific activity in Hepa1c1c7 cells [20]. Some studies have shown that the ingestion of cruciferous vegetables lead to an increase in the activity of ALDH in human saliva and these vegetables were found to be inducers of the enzyme [16,20]. This *in vitro* study reveals that the binding of SF to hsALDH is strong because the binding constant (K_b_) is high i.e 1.23 x 10^7^ M^-1^ (see fluorescence quenching studies). Therefore, it is very likely that SF will bind with the enzyme *in vivo* and lead to its activation.

### Substrate dependent activity assay in the absence/presence of SF

**Fig 3:**
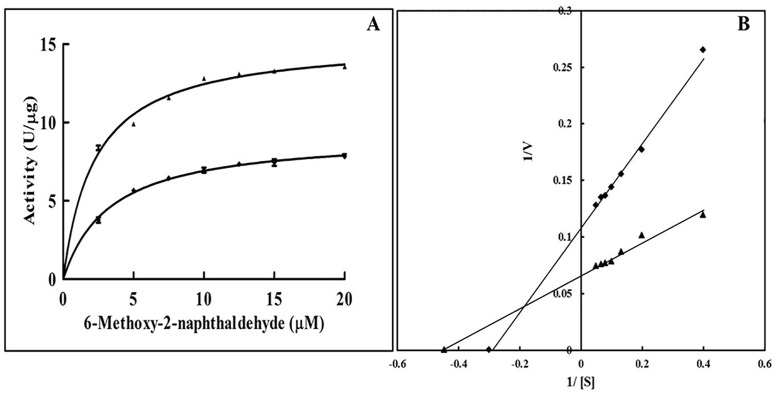
Substrate dependent activity of hsALDH in the absence/presence of SF (A) Michealis Menten plot (B) Line weaver Burk plot. Absence (◆) and presence (▲) of 50 nM SF. Each point represents the mean of two experiments carried out in triplicates.

The activity of hsALDH with varying concentration of substrate (0–20 μM) was determined in the absence and presence of 50 nM SF. The apparent *V*_*max*_ and *K*_*m*_ were calculated by using the Michaelis-Menten plot (Fig 3A) and the Lineweaver-Burk plot (Fig 3B). It was found that the *K*_*m*_ of hsALDH towards the substrate (MONAL-62) decreased and the *V*_*max*_ increased in the presence of SF (Table 1). Therefore, SF increases the affinity of hsALDH for the substrate. SF interacts with the enzyme and favours the binding with the substrate. It is proposed that SF due to its antioxidant property, protects the catalytic Cys 243 amino acid residue from oxidation, which leads to the increase in the catalytic efficiency of the enzyme.

**Table 1:** Kinetic parameters of hsALDH in absence/presence of SF (50 nM).

Kinetic parameters	Without SF	With SF
*V*_*max*_ (U/μg)	9.22 ± 0.18	15.24 ± 0.22
*K*_*m*_ (μg)	3.34 ± 0.25	2.41 ± 0.15

### UV-visible absorption studies

**Fig 4:**
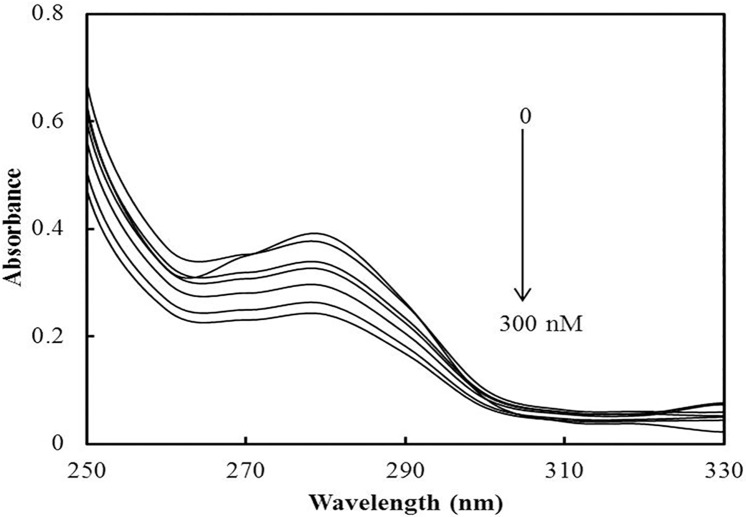
UV absorption spectra of hsALDH in the absence/presence of SF. UV spectra of hsALDH (0.2 mg/ml) in increasing concentration of SF (0–300 nM) were recorded.

UV-visible absorption spectroscopy is an important tool for steady-state studies of protein-ligand and DNA-ligand interaction as well as other biomolecules [37–41]. Changes in the far and near UV regions correspond to the secondary and tertiary structural changes, respectively. In proteins, we can discriminate the various internal chromophoric groups that give rise to electronic absorption bands. The aromatic amino acids contribute to bands in the range of 255–330 nm. Fig 4 shows that the absorption peak of hsALDH centers at 280 nm mainly due to the tryptophan residues. The formation of hsALDH-SF complexes is evident from the spectral data since absorbance decreases with the increase in SF concentration. The shift at 280 nm is not prominent. From these observations we can conclude that SF forms a complex with hsALDH and increases the compactness of the enzyme.

### Fluorescence quenching studies

**Fig 5:**
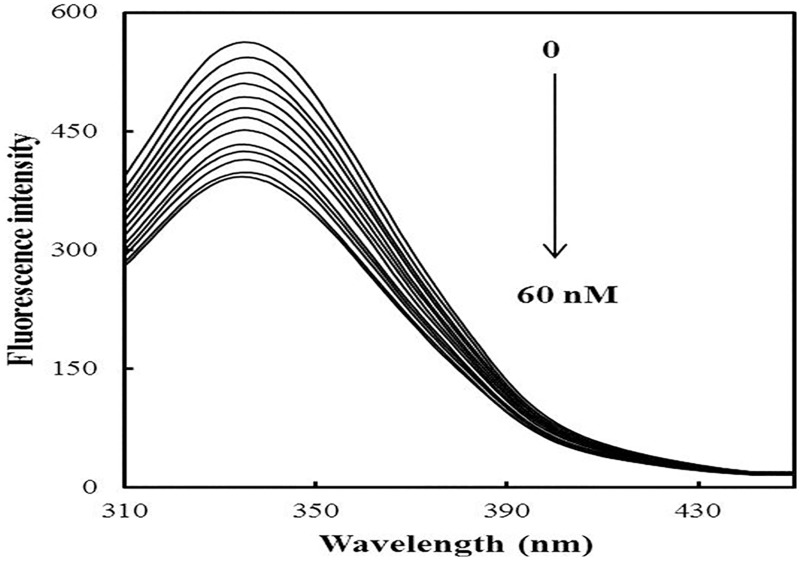
Fluorescence emission spectra of hsALDH in the absence/presence of SF. Spectra of hsALDH (0.2 mg/ml) were recorded in the wavelength range of 300–450 nm in the presence of increasing concentration of SF (0–60 nM).

**Fig 6:**
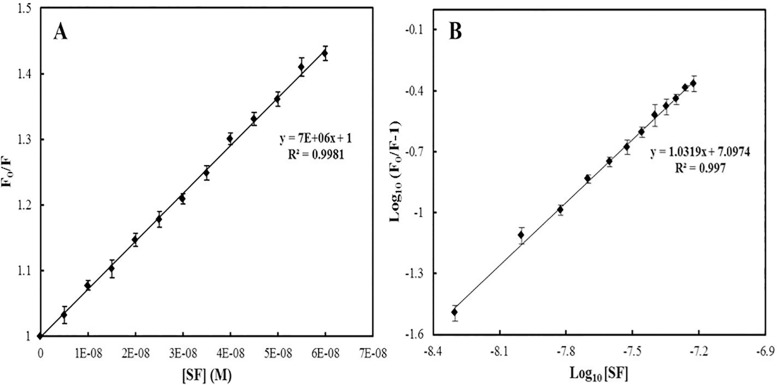
(A) Stern-Volmer plot (B) Modified Stern-Volmer plot for the binding of SF with hsALDH.

Fluorescence measurements give information about the molecular environment in the vicinity of the fluorophore molecules [42–45]. The fluorophores in protein are tryptophan, tyrosine and phenylalanine, however, tryptophan contributes maximally to the fluorescence [46]. Therefore, conformational changes of hsALDH were evaluated by the intrinsic fluorescence intensity before and after the addition of SF. The fluorescence intensity of hsALDH decreased gradually with increasing concentration of SF (Fig 5), which indicated that SF interacts with hsALDH. The decrease in fluorescence intensity upon addition of SF was analyzed according to the Stern-Volmer equation (Fig 6A). There is a linear dependence between F_0_/F and the molar concentration of SF in the Stern-Volmer plot.

There are two types of quenching mechanisms, static and dynamic. In the dynamic mode of quenching, there is collision between ligand and protein which generally increases with increasing temperature, while in the case of static quenching, there is complex formation between ligand and protein which decreases with increasing temperature. When the value of K_q_ is greater than the maximum scatter collision quenching constant (2.0 x 10^10^ mol^-1^ sec^-1^), it shows that quenching is not initiated by dynamic diffusion but occurs by formation of a strong complex between ligand and protein [33,47]. Here, it was observed that the value of K_q_ is greater than that of maximum scatter collision quenching constant (Table 2). Therefore, quenching is not initiated by collision but by complex formation between hsALDH and SF.

**Table 2:** Binding parameters of hsALDH and SF complex.

Complex	K_sv_ (M^-1^)	K_q_ (M^-1^sec^-1^)	K_b_ (M^-1^)	N	R^2^
HsALDH-SF	7 x 10^6^	1.22 x 10^15^	1.23 x 10^7^	1.03	0.997

To determine the binding constant and the number of binding sites, log (Fo/F-1) vs. log [SF] was plotted (Fig 6B). From the slope and intercept of modified Stern-Volmer plots (Eq 3), the number of binding sites and the value of binding constant were calculated. The observed values of K_sv_, K_q_, K_b_ and n are listed in Table 2. Therefore, there is one binding site on hsALDH for SF, and the binding between the two is quite strong.

### Far UV-CD studies

**Fig 7:**
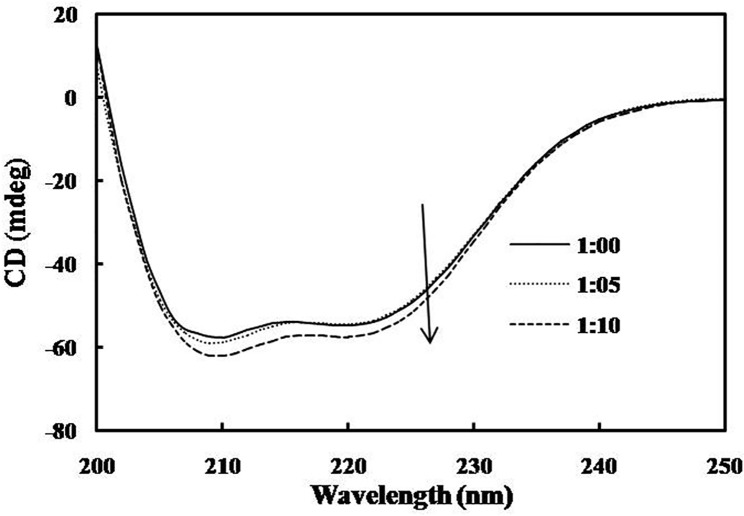
CD spectra of hsALDH in the absence/presence of SF. Far UV-CD spectra of hsALDH (0.2 mg/ml) were recorded with different molar ratios of hsALDH: SF (1: 00, 1: 05 and 1: 10).

CD is an important technique by which the secondary and tertiary structure of biomolecules is studied [48,49]. It also helps in the elucidation of intermediate states like molten globule (MG) during conformational alteration of the proteins [50]. In order to know the conformational changes in hsALDH after interaction with SF, far UV-CD spectra were recorded in absence and presence of SF (Fig 7). The CD spectrum of hsALDH exhibited two negative minima in the UV region at 208 and 222 nm, which is characteristic of the α-helix structure of the protein [51,52]. The binding of SF to hsALDH leads to a small increase in both the negative minima peaks, clearly indicating that the α-helical structure in the enzyme increases to a small extent upon interaction with SF. Further, the CD spectra of hsALDH in the absence and in presence of SF were found to be similar in shape, revealing that the structure of hsALDH is predominantly α-helical even after the addition of SF. Therefore, it appears that the binding of SF with hsALDH stabilizes the native structure of the enzyme without any significant conformational changes. The enzyme appears to become slightly more compact.

### Effect of SF on the activity of hsALDH towards acetaldehyde

**Fig 8:**
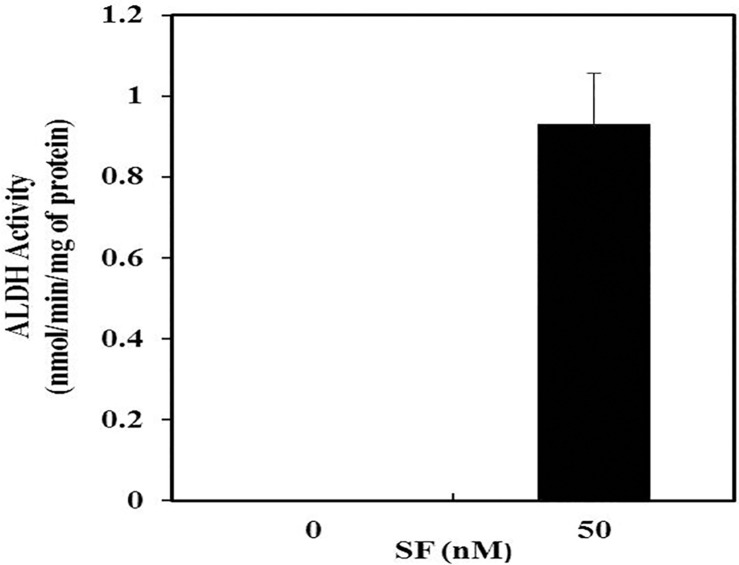
Activity of hsALDH towards acetaldehyde in the absence/presence of SF. The amount of enzyme used in the reaction assay was 20 μg. The concentration of acetaldehyde, NADH and SF used were 50 μM, 100 μM and 50 nM, respectively, and the reaction time was 5 min. The experiment was performed thrice in triplicates.

ALDH2 deficient alcohol consumers are exposed to high concentrations of salivary acetaldehyde and have been found to have an increased risk of upper digestive tract cancers [53]. Also, rinsing with ethanol-containing mouthwashes causes an increase in the acetaldehyde level in the saliva [54]. However, hsALDH is reported to be virtually inactive towards acetaldehyde [29]. Therefore, we examined whether SF can also activate hsALDH to oxidize acetaldehyde. The activity of hsALDH towards acetaldehyde as the substrate in the absence and presence of SF was studied (Fig 8). In the absence of SF, hsALDH showed virtually no activity towards acetaldehyde. However, in the presence of 50 nM SF, the enzyme exhibited significant activity towards acetaldehyde. Therefore, it is expected that SF should protect individuals from salivary acetaldehyde induced toxicity by activating hsALDH to oxidize acetaldehyde.

### Molecular docking analysis

**Fig 9:**
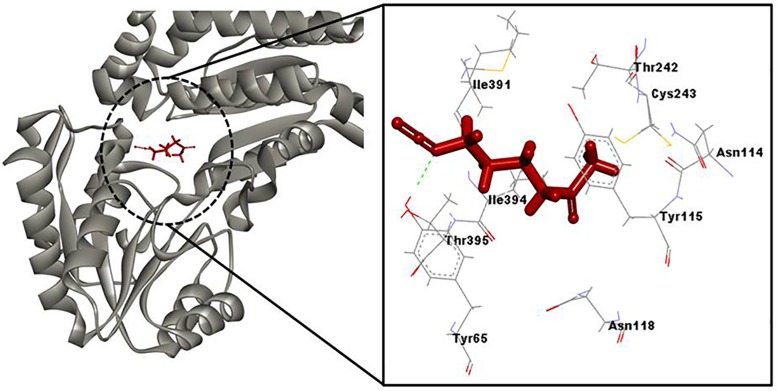
Docking structure. (A) Molecular docking structure of hsALDH and SF (B) Amino acid residues interacting with SF.

The docking results clearly showed that SF binds to the active site cavity of the ALDH3A1 and predominantly interacts with Ile 391, Thr 242, Tyr 115, Asn 114, Cys 243 and Tyr 65 (Fig 9A and 9B). The amino acid residues of the enzyme interact with SF mainly through hydrophobic interactions and hydrogen bonding. Tyr 65 is hydrogen bonded with SF while Ile 391, Thr 242 and Tyr 115 interact with SF through hydrophobic interactions. SF binds near the Cys 243 residue which acts as a nucleophile and plays a major role in the reaction catalyzed by the enzyme. It is very likely that SF due to its antioxidant property, protects the catalytic Cys 243 residue from oxidation, which leads to the increase in the catalytic efficiency and hence the activation of the enzyme. Also, SF interacts with Asn 114 which is a highly conserved residue in the catalytic domain of ALDHs and is having a role in the stabilization of oxyanion form of the thiohemiacetal during catalysis [55]. Therefore, SF fits inside the catalytic center and occupies some portion of the active site and thus decreases the overall size of active site cavity. We speculate that the reduced cavity now becomes capable of fitting the small acetaldehyde molecule, and therefore hsALDH starts catalyzing it.

## Conclusions

The present study clearly shows that the cruciferous vegetables and their key bio-active compound SF activate hsALDH which is a very important detoxifying enzyme in the mouth. UV absorbance, fluorescence and CD studies revealed that SF binds to hsALDH and does not disrupt its native structure. The thermodynamic parameters indicate the formation of a spontaneous strong complex between hsALDH and SF. SF interacts with the enzyme and increases its affinity for the substrate. Molecular docking analysis revealed that SF occupies some portion of the active site of the enzyme and interacts with the important amino acid residues present in it, including the catalytic Cys 243 which acts as a nucleophile during catalysis. We propose that SF due to its antioxidant property, protects the catalytic Cys 243 residue from oxidation, which leads to the increase in the catalytic efficiency and hence the activation of the enzyme. In addition, the enzyme showed significant activity towards acetaldehyde in the presence of SF and hence can protect individuals who are alcohol intolerant against acetaldehyde toxicity. It is therefore very likely that consumption of large quantities of cruciferous vegetables or SF supplements can lower the risk of acetaldehyde mediated toxicity and oral cancer development.
